# Idol Worship: How Does It Influence Fan Consumers’ Brand Loyalty?

**DOI:** 10.3389/fpsyg.2022.850670

**Published:** 2022-04-27

**Authors:** Libin Chen, Guanhong Chen, Shuxu Ma, Shuo Wang

**Affiliations:** Business School, Beijing Technology and Business University, Beijing, China

**Keywords:** idol worship, brand personality appeal, brand-based self-realization, perceived emotional value, relatedness needs satisfaction, brand loyalty

## Abstract

The brand loyalty of fan consumers can be influenced by idol worship if certain celebrity figures are linked with a brand. Collaborating with idols is an effective marketing strategy that many companies use to enhance their appeal to fan consumers and increase their brand equity. Fan consumers demonstrate passion and admiration for their idols, and this psychological phenomenon affects their cognition of brands that are endorsed by their favorite celebrity figures. The purpose of this study was to explore the influence mechanism that propels fan consumers’ brand loyalty and the mediating effects of brand passion and brand attachment. Our results revealed the following key findings: (1) brand personality attraction, perceived emotional value, brand-based self-realization, and relatedness needs satisfaction have a significant effect on brand passion; (2) perceived emotional value and relatedness needs satisfaction have a significant effect on brand passion attachment; (3) brand passion can directly affect brand loyalty, but it also indirectly affects brand loyalty through brand attachment; (4) brand personality appeal, brand-based self-realization, and relatedness needs satisfaction can influence brand attachment through brand passion and ultimately have an impact on brand loyalty; (5) brand perceived emotional value and relatedness needs satisfaction affect brand loyalty through brand attachment. These findings have several implications for enterprises that want to meet fan consumers’ emotional needs, enhance brand loyalty through the use of idol brand endorsement, or implement brand campaigns that involve idols.

## Introduction

Fan consumption has become a large part of today’s market. Many fan consumers are influenced by their idols when choosing products. Due to idolatry, they would like to purchase the brands endorsed by their idols. Idol worship refers to psychological identification with and emotional attachment to an idol figure (Cheung and Yue, 2012). Previous research has suggested that idolatry positively affects an individual’s preference for certain products and their intention to buy (Wang et al., 2009). Consequently, cooperation with idols has been regarded as an effective means enhancing a brand’s image and influencing consumer attitudes (Singh and Banerjee, 2019). Using idol worship to strengthen the positive impression a particular brand makes in the minds of consumers and encourages buyers to choose that brand has become an important marketing strategy for many companies.

There is a growing consensus that the long-term success of a brand is dependent on building and sustaining strong brand loyalty, and many brands continuously seek out ways to enhance loyalty as a means of increasing their profits (Swaminathan et al., 2018). Brand loyalty refers to the tendency of consumers to continue to repurchase the same brand and to become loyal with a deep commitment toward the brand (Oliver, 1993). Companies build brand loyalty in various ways, such as offering a high-quality product, providing a quality customer experience, or building up the unique style or personality of their brand. Some enterprises also use celebrity endorsement to develop the connection between their brand and consumers (Dwivedi et al., 2015), which plays an important role in enhancing brand loyalty. These celebrities are often prominent figures working in specific fields, and they usually have a large number of fans. They are also the idols that fans tend to worship. Compared with other regular celebrities, idols receive a notable display of enthusiasm from their fans (Zhao and Wu, 2020). The relationship between fans and their idols is considered to be a more intimate connection, but previous studies have rarely explored how this particular relationship affects fan consumers’ brand loyalty.

Research in the field of brand endorsement suggests that companies generally choose celebrities who align with the personality of their brand and that this approach increases the attractiveness of the brand (Pradhan et al., 2016; Zamudio, 2016). Previous research has pointed out that celebrity endorsement may bring brand awareness, reputation, expertise, trustworthiness, and liking to a brand (Hoekman and Bosmans, 2010; Nelson and Deborah, 2017). McCracken (1989) argues that a celebrity comes with a whole constellation of meaning (e.g., lifestyle, status, and trendiness), which are being transferred to the brand during the endorsement process. As a symbol carrying meaning, the brand possesses the characteristics of the endorser. If the endorser of a brand is an idol of the consumers, the brand then possesses a collection of meanings of their idol.

There is a special emotional bond between fans and their idols, with a more intimate relationship, and the idol usually embody the ideal self-concept for the fans. This particular relationship could have an impact on fan consumers’ attitudes and loyalty to the brand. Psychologically, idolatry involves the formation of an emotional attachment between fans and their idols (Yue and Cheung, 2000), which indicates that the relationship between fans and their idols has an emotional meaning. When idols wear or use products from a certain brand, fan consumers try to imitate them for self-improvement purposes. In this way, a brand can allow fan consumers to experience self-actualization and self-improvement (Mukherjee, 2009). When companies work with celebrities, fans can get closer to their idols by purchasing from endorsed brands and gaining a sense of satisfaction from having met their relationship needs (Thomson, 2006).

In summary, idol endorsement may affect the brand loyalty of fan consumers from four aspects: (1) brand personality appeal, (2) perceived emotional value, (3) brand-based self-realization, and (4) relatedness needs satisfaction. In what follows, we explore whether the emotional attachment between fans and idols affects the relationship between fans and idol-endorsed brands. By examining the role that idol worship plays in this process, our study contributes to a broader understanding of brand loyalty and offers practical implications for brand marketers seeking efficient ways to enhance consumer-brand relationships. In this study, we depend on literature research to summarize the influence of idolatry on fan consumers in brand consumption and use empirical research to explore the influence mechanism.

## Theoretical Background and Hypotheses

### Theoretical Background

Social identity theory attempted to explain when and why individuals identify with groups and act as part of them (Zeugner-Roth et al., 2015). Stets and Burke (2000) pointed out that social identity is considered to be an individual’s perception and awareness of the group or social category to which he or she belongs. Social identity theory posits that people tend to place themselves into specific social categories after undergoing a cognitive categorization process that involves finding similarities and overlaps with a compared group (Bi et al., 2018). This identity derives largely from favorable comparisons between the own group (in-group) and other groups (out-groups) and shows more liking to in-groups (Bochatay et al., 2019). Social identity theory also claims that creating and maintaining identity becomes an individual’s primary motivation for participating in social activities (White, 2010) and that this influences behavior. In this context, companies that work with idols promote specific brands and encourage fan customers to build a strong sense of identification and positive emotional connection with their idols. Fan customers may develop a sense of belonging to its community by confirming their true loyalty through participation in the group (Kuo and Hou, 2017). This link between idols and brands may foster brand passion, which could then translate into consumer loyalty (Swimberghe et al., 2014).

Bowlby (1969) assumes that attachment is an emotionally charged connection between people. Idol attachment can be defined as an emotional bond between an individual and a human brand (Huang et al., 2015). This association will lead to a tendency to make commitments, investments, and sacrifices to the attachment object, all of which can indicate the extent to which the consumer seeks to maintain a long-lasting relationship with the attachment object. Therefore, consumers pour more resources into the idols they admire to express their feelings in this way. Idol attachment might lead consumers to purchase products connected to their idols (Huang et al., 2015) and fulfill experiential, symbolic and emotional needs (Park et al., 2006). Companies that integrate elements associated with idols into their branding can thus kindle positive emotions and purchase intention in fans (Aw and Labrecque, 2020). Gradually, this dynamic might stimulate the emotional migration of fan consumers (Ilicic and Webster, 2014). This emotional migration allows companies to extend the attachment that fan consumers have to their idols to a certain brand, which may encourage them to develop brand loyalty (Bıçakcıoğlu et al., 2018).

In addition to the above two theoretical foundations, scholars have explained the mechanism of idolatry from the perspectives of self-actualization and relational needs. Self-actualization refers to an individual’s desire to continuously stimulate his or her own potential and realize personal pursuits (Hoffman, 2020). It is a state of mind that is commonly possessed by human beings. When individuals feel more like or closer to their favorite idols, the need for self-actualization is subsequently satisfied to some degree (Albert et al., 2017). Previous research suggested that relatedness needs are the need to belong or desire for interpersonal attachments, which have been regarded as fundamental psychological needs or human motivation (Baumeister and Leary, 1995; Hong et al., 2019). Consumers form strong emotional and psychological ties with their idols by purchasing the brands they endorse, thus gaining a sense of belonging satisfaction (Escalas and Bettman, 2017).

### Hypotheses

#### Brand Personality Appeal

Freling et al. (2011) introduced the concept of brand personality appeal and defined it as a brand’s ability to attract consumers through its personality traits. Compared with the measurement of brand personality, brand personality appeal focuses on consumer perceptions of brand personality (Gordon et al., 2016). It determines whether a brand creates consumer brand personality perceptions and predicts consumer reactions to a brand’s personality. Freling et al. (2011) classified brand personality appeal into three dimensions: favorability, originality, and clarity. Favorability refers to the extent to which consumers hold a positive view of the brand’s personality. When consumers perceive the brand personality as being beneficial to them, it enhances their satisfaction with the brand and results in a positive evaluation. Originality refers to the extent to which consumers perceive a brand’s personality to be novel and distinct from other brands in the same product category. When consumers perceive the brand personality as being unique, it helps them distinguish it from other brands in the market. Clarity refers to the extent to which a brand’s personality is apparent and recognizable to consumers. In other words, clarity is linked to whether or not a brand’s personality is identifiably beneficial or unique to a customer. Scholars have further supported the utility of this concept and model through relevant empirical studies (Freling et al., 2011; Gordon et al., 2016; Tho et al., 2016). Under the influence of idol worship, brands build and deliver brand personality by cooperating with idols, and their brand personality can create a corresponding appeal to fan consumers.

Brand passion refers to a primarily affective, extremely positive attitude toward a specific brand that leads to emotional attachment and influences relevant behavioral factors (Bauer et al., 2007), which describes the zeal and enthusiasm features of consumer-brand relationships (Keh et al., 2007). For consumers, brand passion acts as a motive for buying or owning a particular brand (Albert et al., 2017). Ambroise et al. (2014) suggested that the endorser of the brand is one vital source of brand personality. Based on social identity theory, incorporating an idol’s personality and image into a brand helps express the brand personality that matches the idol and can give the brand the identity of a specific in-group. This identity heightens a fan consumer’s identification with the brand and creates a unique brand personality appeal to fan consumers. Ultimately, this dynamic causes fan consumers to transfer their fascination with their idols to a desire for the brand (Hung et al., 2011), thus promoting brand passion. Therefore, this study proposes the following hypothesis:

H1: Brand personality appeal is positively related to brand passion.

Brand attachment is the strong emotional connection that exists between a consumer and a brand. A consumer’s emotions are based on their perception of a brand’s personality and image (Chen, 2019). Therefore, brand personality building is likely to trigger brand attachment. By incorporating elements of an idol, a brand can obtain distinct personality traits that are appealing to consumers and offer them a unique and meaningful brand experience. These experiences encourage consumers to form positive judgments about a particular brand personality and establish a strong connection with a certain brand. In this way, the brand becomes important to the consumer, and switching brands could lead to a loss of meaning, which enhances emotional attachment (Fullerton, 2005), thus enhancing emotional attachment. Accordingly, brand personality is an important factor that influences the brand attachment of fan consumers, and it causes consumers to develop an emotional attachment to certain brands. Therefore, this study proposes the following hypothesis:

H2: Brand personality appeal is positively related to brand attachment.

#### Perceived Emotional Value

Consumers may draw emotional value from the brand, where utility is derived from feelings toward the brand (Sweeney and Soutar, 2001). Emotional value is derived from a mix of sources, including relational sources, as well as pleasure and enjoyment sources (France et al., 2020). The generation of these sources helps enhance the perceived emotional value of the brand, and the source of the relationship is a reflection of the relationship in the emotional value. Idolatry is a pseudo-social relationship. By cooperating with idolized celebrity figures, brands are satisfying the relationship needs that exist between consumers and their idols. An additional source of relationship value can be attained for the brand, thus enhancing its perceived emotional value.

In fact, the emotional connection between consumers and brands has a significant impact on brand purchasing behavior, so evoking emotions has become an important brand management approach. When consumers interact with a brand, brands that purposefully can certain emotions are more likely to make consumers associate their emotional responses with the brand, which enhances the customer’s perception of the brand’s emotional value (Parmar and Mann, 2021). Although previous researches have not empirically verified that there is an emotion transfer between the endorser and the brand. McCracken (1989) has suggested that there is a meaning transfer from endorser to the brand. Consumers become emotionally connected to the brand as the brand carries the meaning of their idols. Therefore, companies that integrate idols into their brands can stimulate the emotional migration of fan consumers (Ilicic and Webster, 2014), enhance their recognition of the brand and its emotional value, and kindle positive emotions (Bai et al., 2021). The perceived emotional value of the brand interacts with the consumer’s brand experience to ultimately produce brand passion. Therefore, this study proposes the following hypothesis:

H3: Perceived emotional value is positively related to brand passion.

As previously mentioned, the perceived emotional value of a brand originates in how consumers feel about the brand, and companies that collaborate with idols heighten fan consumers’ positive perception of their brand. According to emotional attachment theory, when people experience stress in external environments, they often seek physical or psychological protection from an attachment object. For fans, idols are attachment objects. Brands that work with idols offer unique meaning to fan consumers, which encourages them to partake in emotional experiences with the brand. This dynamic also increases the perceived emotional value and positive cognition of the brand. Moreover, research has shown that positive cognition of brands significantly contributes to brand attachment (Wu et al., 2017). Therefore, this study proposes the following hypothesis:

H4: Perceived emotional value is positively related to brand attachment.

#### Brand-Based Self-Realization

Waterman (1993) suggests that the theory of self-realization derives from realization theory, in which individuals can experience a sense of self-realization by striving for excellence and realizing their potential. Raibley (2012) also argues that a sense of self-realization arises when a person achieves their objectives and further emphasizes that its core lies in the pursuit of self-value. In this process, self-realization is accompanied by the individual’s continuous self-identification. Previous research also insisted that self-realization involves continually recognizing the self and gradually realizing one’s potential (Clark, 2018). In this process, the individual continuously strives toward the ideal self and recognizes the essence of self through identification, thus enhancing the self and generating a sense of self-actualization.

According to identity theory, brands are essential to individual self-expression. The experience that the brand brings to the consumer encourages the consumer to embrace the brand. The stimulus related to self-representation makes the consumer view their identification with the brand as part of their own identity (Das et al., 2019). Brands that are associated with idols appeal to fans because this link fulfills their internal expectations of self-realization, helps affirm their fan identity (Huang et al., 1993; Zhang and Hung, 2020), or allows them to get closer to their ideal self by emulating their idols. Consequently, fans buy and use products and services from brands that are associated with their idols to affirm themselves, which is a process that reflects a customer’s internalization of a brand. Brand passion refers to the strong emotions of individuals who desire to engage with a brand and integrate it into their identity (Swimberghe et al., 2014), which essentially reflects the internalization of the brand (Kim et al., 2020). The satisfaction of brand-based self-realization can lead consumers to internalize the brand, which further stimulates individual brand passion. Therefore, this study proposes the following hypothesis:

H5: Brand-based self-realization is positively related to brand passion.

Research has shown that a person becomes attached to an object because some features of the object have the function of self-realization or self-expression (Chen, 2019), so a brand that has the function of self-realization can promote the emergence of brand attachment (Donvito et al., 2020). According to social identity theory, people define their self-concept through their association with social groups or organizations and present their group identity through their actions. Being a fan is an identity, and individuals often purchase and use products related to the idols that they admire to highlight this identity. Thus, when a brand collaborates with idols, the fans who admire them will develop a desire to buy the brand. By making contact or forming a connection with brands that work with idols, fans can further highlight or validate their identity. In this way, they can experience a sense of self-realization based on the brand and form an attachment to the brand. Therefore, this study proposes the following hypothesis:

H6: Brand-based self-realization is positively related to brand attachment.

#### Relatedness Needs Satisfaction

Relatedness needs satisfaction refers to an individual’s sense of intimacy and belonging and their desire to connect with and be cared for by others (Baumeister and Leary, 1995; Ryan, 1995; Moller et al., 2010; Jiang et al., 2018). According to interpersonal theory and the belongingness hypothesis, people have a need to belong and a tendency to form and maintain social relationships and emotional connections (Hong et al., 2019), which shows that the satisfaction of relational needs has an important impact on the individual. Self-determination theory further suggests that relatedness needs, together with autonomy or competence needs, constitute the basic psychological needs of human beings. According to this theory, the need for autonomy reflects the need for self-willed choice in the performance of an activity. Competence needs include the need to feel satisfied with the achievement of objectives and the effective implementation of activities. Relatedness needs are related to building intimacy, experiencing a sense of belonging to a community and feeling part of a group (Ryan and Deci, 2000). Research shows that consumer satisfaction is shaped by the fulfillment of associative needs, which motivates individual behavior and can predict individual behavioral performance. When relatedness needs are met, it provides the emotional security required to create strong attachments (Gilal et al., 2020; Kim et al., 2020). In the context of this study, the desire of fans to connect with the figures that they admire reflects their need to create and maintain a relationship with their idols, and brands that work with celebrities can strengthen this link to some extent.

Brand passion is considered to be a motive for owning a particular brand (Albert et al., 2017), and relatedness needs satisfaction can help trigger this motivation. Specifically, fans try to build a lasting and intimate relationship with their idol in their daily lives. This process involves familiarizing themselves with their idol through different media sources, community participation, and other methods that allow them to satisfy their relatedness needs. These activities allow fans to relate to the celebrities that they admire and become passionate about their interests (Kim et al., 2020). Therefore, when a fan customer perceives that a brand can narrow the distance between them and their idol, the brand will benefit from this association. Specifically, the consumer’s perceived association with the brand can provide fans with a sense of connection to their idols, which can improve the satisfaction of consumers’ relatedness needs to a certain extent. When consumers engage with a brand, some stimuli and cues associated with the brand can prompt them to internalize the brand when they meet a specific need, thus triggering brand passion (Das et al., 2019). The relatedness needs satisfaction can thus have an impact on a consumer’s passion for the brand. Therefore, this study proposes the following hypothesis:

H7: Relatedness needs satisfaction is positively related to brand passion.

Relatedness needs are a type of basic psychological desire that all people experience. When relatedness needs are met, it provides the emotional security required to create strong attachments (Gilal et al., 2020; Kim et al., 2020). Brand-idol cooperation satisfies a fan consumer’s desire to foster intimacy with their idol and promotes positive emotions through their admiration for certain celebrities (Ilicic et al., 2016). Based on the belongingness hypothesis, consumers that have a high-need to belong purchase idol-endorsed brand products to further enhance their sense of belonging. This dynamic causes the consumer to feel more connected to the brand and develop positive emotional meanings that continuously influence their feelings. Over time, this emotional response gradually transforms into a more lasting emotional attachment (Vredeveld, 2018). Thus, relatedness needs satisfaction that is triggered by a brand can create an emotional connection between the consumer and the brand, which enhances brand attachment. Therefore, this study proposes the following hypothesis:

H8: Relatedness needs satisfaction is positively related to brand attachment.

#### The Mediating Role of Brand Passion and Brand Attachment

In recent years, brand passion as a critical factor in building and enhancing brand loyalty has drawn the attention of researchers working in brand-consumer relationship studies (Kim et al., 2020). Brand passion often leads to positive, passion-driven behaviors (Batra et al., 2012), which makes it the most important component of brand management. Meanwhile, in some cases, consumers tend to remain committed to a brand when they anticipate being troubled by the absence of that brand (Rauschnabel and Ahuvia, 2014). Shimp and Madden (1988) argue that brand loyalty is equivalent to having a perfect love for a brand that involves preference, passion, and commitment. Therefore, when brands trigger passion in their consumers, they will be loved and have a loyal base. Furthermore, previous research on the consequences of brand passion has found that consumer passion for a brand can translate into consumer loyalty (Swimberghe et al., 2014). Therefore, this study proposes the following hypothesis:

H9: Brand passion is positively related to brand loyalty.

Brand attachment is an important factor that influences brand loyalty. Attachment to a brand can make consumers more willing to spend money or try to obtain products or services from a certain brand. Customers also recommend the brand and use word of mouth to encourage other consumers to repurchase products and services (Kwon and Mattila, 2015). In other words, when consumers develop a strong emotional connection to a brand, they become less sensitive to brand price increases and are more likely to buy the same brand repeatedly (Kim et al., 2020). Moreover, studies have proven that consumers personify brands and develop different types of relationships with them. Strong brand relationship quality means high levels of loyalty in customers. To attract loyal customers, brands need to develop a strong love or attachment relationship with their consumers (Rohra and Sharma, 2016). Researchers have also claimed that consumers with stronger emotional bonds to a brand might feel satisfied and committed to it and thus be more inclined to be loyal (Bıçakcıoğlu et al., 2018). Therefore, this study proposes the following hypothesis:

H10: Brand attachment is positively related to brand loyalty.

As consumers come to know more about the brand, they form a corresponding association with the brand’s image and personality. Consumers’ positive associations trigger their positive emotions toward the brand, and their emotional state toward the brand affects their level of association with it. In other words, when consumers experience positive emotions over a brand, those feelings promote a strong connection. Brand passion is defined as a primarily affective and extremely positive attitude toward a specific brand (Yalntekin and Saygl, 2020). Brand passion can stimulate strong emotions and give further meaning to the consumer-brand relationship. It also motivates customers to invest resources, form a strong connection with the brand, and remain loyal over time (Das et al., 2019). Finally, brand passion causes emotional attachment to a certain brand and can kindle consumer intimacy and enthusiasm for a brand (Kim et al., 2020). Therefore, this study proposes the following hypothesis:

H11: Brand passion is positively related to brand attachment.

**FIGURE 1:**
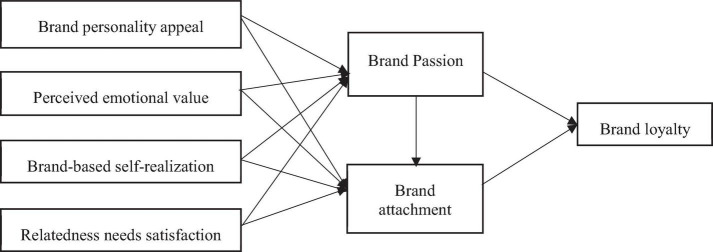
Research model.

Based on the discussion above about each variable and their related theories, the influence of idolatry on consumers’ brand perceptions and emotions is taken as a starting point in this study. We propose four influencing factors: (1) brand personality attraction, (2) perceived emotional value, (3) brand-based self-realization, and (4) relatedness needs satisfaction. These factors can stimulate consumers’ brand passion and brand attachment to idol-related brands and ultimately lead to the stimulation of brand loyalty. Based on above discussion, the following specific empirical research model was constructed (see Figure 1).

## Materials and Methods

### Variable Measurement

In order to ensure that the seven variables involved in this study can be measured effectively, the scales of each variable were obtained by reading the relevant literature and drawing on the relatively mature scales. In addition, in order to better fit the characteristics of this study, the variable items were modified according to the content of this study. The measures in this study were assessed using a 7-point Likert scale.

#### Brand Personality Appeal

Brand personality appeal was measured based on a scale proposed by Tho et al. (2016). The scale consists of three dimensions-favorability, originality, and clarity-and includes three items. The exemplary item of favorability was “the brand has a very attractive personality compared to other brands.” The item for originality was “the brand’s personality is distinctive compared to other brands” and the item for clarity was “the personality of the brand is easily recognizable compared to other brands.” Participants rated each item using a 7-point Likert scale (ranging from 1 = “strongly disagree” to 7 = “strongly agree”). Higher scores indicated higher levels of brand personality appeal.

#### Perceived Emotional Value

Perceived emotional value was assessed using a scale created by Hou et al. (2020). The perceived emotional value comprises five items and the exemplary items of the scale were “compared to other brands, this brand makes me feel better.” Participants used a 7-point Likert scale (ranging from 1 = “strongly disagree” to 7 = “strongly agree”) to indicate what they thought about brands that they had purchased from in the past because of idolatry. Higher scores indicated higher levels of perceived emotional value.

#### Brand-Based Self-Realization

Brand-based self-realization was measured using a scale designed by Waterman (1993) and Mandil (2016). The brand-based self-realization scale includes four items, such as “having the brand gives me a greater sense of fulfillment and satisfaction.” Participants rated each item using a 7-point Likert scale (ranging from 1 = “strongly disagree” to 7 = “strongly agree”). Higher scores indicated higher levels of brand-based self-realization.

#### Relatedness Needs Satisfaction

Relatedness needs satisfaction was assessed using a scale created by Chen et al. (2015) and Gilal et al. (2020). The relatedness needs satisfaction scale includes four items such as “I think I have a connection with the brand.” Participants used a 7-point Likert scale (ranging from 1 = “strongly disagree” to 7 = “strongly agree”) to indicate what they thought about brands that they had purchased from in the past because of idolatry. Higher scores indicated higher levels of relatedness needs satisfaction.

#### Brand Passion

Brand passion was measured using a scale adapted from the work of Kim et al. (2020) and Pourazad et al. (2020). The brand passion scale includes five items, such as “I was very excited to see the brand.” Participants rated each item using a 7-point Likert scale (ranging from 1 = “strongly disagree” to 7 = “strongly agree”). Higher scores indicated higher levels of brand passion.

#### Brand Attachment

Brand attachment was assessed using a scale created by Japutra et al. (2018) and Garanti (2019). The brand attachment scale includes six items, such as “I feel attached to the brand.” Participants rated each item using a 7-point Likert scale (ranging from 1 = “strongly disagree” to 7 = “strongly agree”). Higher scores indicated higher levels of brand attachment.

#### Brand Loyalty

Brand loyalty was assessed using a scale created by Hwang et al. (2019). The brand loyalty scale includes four items, such as “I intend to visit more often in the future.” Participants rated each item using a 7-point Likert scale (ranging from 1 = “strongly disagree” to 7 = “strongly agree”). Higher scores indicated higher levels of brand loyalty.

### Participants

The data was collected through a questionnaire survey, which consisted of two parts. The first measured the variables related to a brand purchase made by fan consumers under the influence of idolatry. The second collected basic information about the consumers, such as gender, age, occupation, education, and income. The scale is listed in Appendix A. We delivered the questionnaire through social media to college students and individuals who work at enterprises. Participants were asked to recall instances when they bought from certain brands because of their idolatry. They then answered the questionnaire based on the actual thoughts that they had during the purchasing process. A total of 388 questionnaires were distributed during the study period and 332 valid questionnaires were obtained after eliminating the questionnaires with missing values or contradictory answers.

The majority of the participants were female (216 [65.1%] female and 116 [34.9%] male). About 227 (68.4%) participants in this study were between the ages of 18 and 25, 58 (17.5%) were aged between 26 and 30, 27 (8.1%) were aged between 31 and 40, 10 (3%) were between 41 and 50, and 7 (2.1%) were between 51 and 60. Most of the participants were students (153, 46.1%), 68 (20.5%) were general staff, 40 (12%) were professional, 33 (9.9%) were teachers, 23 (6.9%) were members of management, and 15 (4.5%) belonged to other careers. We also found that 118 (35.5%) of the participants had a monthly income between 632 USD and 1,265 USD, 97 (29.2%) had a monthly income of under 158 USD, 85 (25.6%) had a monthly income between 158 USD and 632 USD, and 32 (9.5%) of the participants had a monthly income more than 1265 USD.

### Methods

We used structural equation modeling (SEM) in AMOS to examine the interrelationships between the study variables and test the mediation hypothesis. The SEM analysis was predominantly confirmatory because the theoretical rationale was regarded as important, and the goal was to provide a quantitative evaluation of the hypothesized theoretical model. In the model, brand personality appeal, perceived emotional value, brand-based self-realization, and relatedness needs satisfaction were independent variables. Brand passion and brand attachment were mediation variables, and brand loyalty was a dependent variable.

## Results

### Descriptive Statistics and Correlation

The indicators for all of the variables are displayed in Table 1. The descriptive analysis showed that all the variables had good scores for symmetry and kurtosis. Furthermore, correlation analysis also showed that all the variables are positively correlated with each other.

**TABLE 1:** Descriptive statistics and correlation among variables.

	Min	Max	*M*	*SD*	Skew	Kurt	1	2	3	4	5	6
Brand personality appeal	1.00	7.00	5.22	1.12	1.150	2.097						
Perceived emotional value	2.17	7.00	5.28	0.95	0.764	0.421	0.443**					
Brand-based self-realization	2.00	7.00	5.05	0.95	0.498	0.158	0.459**	0.618**				
Relatedness needs satisfaction	1.00	7.00	4.98	1.18	0.601	0.004	0.399**	0.523**	0.652**			
Brand passion	1.50	7.00	5.13	1.10	0.816	0.508	0.492**	0.535**	0.669**	0.597**		
Brand attachment	1.50	7.00	4.88	1.07	0.586	0.153	0.405**	0.535**	0.625**	0.641**	0.670**	
Brand loyalty	1.50	7.00	5.28	1.02	0.700	0.620	0.470**	0.610**	0.561**	0.506**	0.624**	0.664**

N = 332; α, Cronbach’s alpha; M, Mean; SD, standard deviation; Skew, skewness; Kurt, kurtosis; **p < 0.01.

### Measurement Model

#### Measurement Evaluation

The explanatory factor analysis (EFA) was performed to examine the internal and external reliability of the constructs. In particular, the instruments’ Cronbach alpha (α), factor loadings, composite reliability (CR), and average variance extracted (AVE) were computed. All scores obtained during the EFA were recorded above the least acceptable value, as shown in Table 2. Moreover, the satisfactory results were also computed in the case of each subgroup.

**TABLE 2:** Internal reliability testing for the overall collected sample.

Construct	Items	Loadings	α	CR	AVE
Brand personality appeal	3	0.833–0.821	0.872	0.873	0.695
Perceived emotional value	5	0.718–0.769	0.883	0.898	0.595
Brand-based self-realization	4	0.514–0.745	0.850	0.872	0.578
Relatedness needs satisfaction	4	0.691–0.839	0.911	0.911	0.720
Brand passion	5	0.642–0.774	0.908	0.921	0.664
Brand attachment	6	0.586–0.726	0.883	0.875	0.540
Brand loyalty	4	0.586–0.788	0.898	0.900	0.693

*Authors calculations. α, Cronbach alpha; CR, composite reliability; AVE, average variance extracted.*

Validity analysis usually consists of two parts: content validity and construct validity. The scales in this study are derived from established foreign scales and therefore have good content validity. In this study, KMO value is 0.952 and Barlett’s sphericity test p-value is 0.000. The data in this study are suitable for factor analysis. Common factors were extracted by the principal component method and applies orthogonal rotation to the factor loading array as a way to obtain the cumulative total explained variance and factor loadings. The cumulative variance explained by these seven common factors was 73.44%. The factor loading coefficients for all question items in Table 2 are greater than 0.5. It indicated that the questionnaire has good construct validity.

Common Method Bias Test (CMBT), also known as homoscedasticity, is often caused by the fact that the data in questionnaires are derived from the same measurement instrument and the variable scores are all derived from the same measurement object. In this study, Harman’s one-factor test was used to test the data of CMBT.

In this study, the 31 question items of the 7 constructs of the full sample (*N* = 332) data were tested together. A total cumulative explained variance rate of 73.44% and a first-factor variance explained the rate of 12.331%, which is below 40%. These results all indicate that the common method bias is not severe enough to warrant special treatment in the subsequent analysis.

Meanwhile, multicollinearity was tested by computing the variance inflation factor (VIF) for each item of the proposed constructs. All observed items’ VIFs were lower than the upper cutoff limit, as recommended by Petter et al. (2007).

#### Goodness of Fit Statistics

**FIGURE 2:**
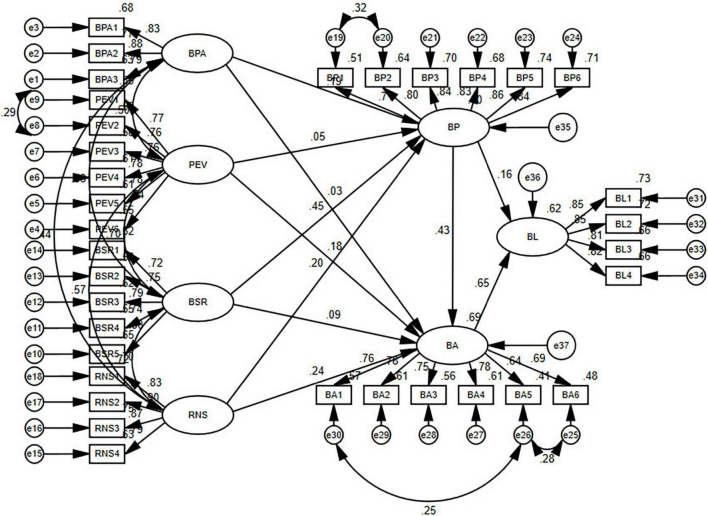
Structural model. BPA, brand personality appeal; PEV, perceived emotional value; BSR, brand-based self-realization; RNS, relatedness needs satisfaction; BP, brand passion; BA, brand attachment; BL, brand loyalty.

This study used confirmatory factor analysis (CFA) to conform to the standards of convergent validity. Furthermore, to test the statistical model’s goodness of fit, we selected some fit metrics (Table 3). The results of CFA show that the incremental fit index (IFI) was 0.981, that the comparative fit index (CFI) was 0.953, and that the Tucker–Lewis index (TLI) was 0.974. All of the fitness indices are at the required level. The value of RMSEA (root mean square error approximation) was 0.049, which was also within an acceptable range. In addition, the results of the structural model are shown in Figure 2.

**TABLE 3:** Model fit statistics.

Goodness of fit indices	Norms	Structural model
χ^2^/df	>1 and <5*	1.802
IFI	≥0.90*	0.953
CFI	≥0.90*	0.953
TLI	≥0.90*	0.947
RMSEA	<0.05*	0.049

*Recommended limits by Hu and Bentler (1999).

### Path Analysis

After completing the confirmatory factor analysis, we conducted the path analysis and tested the structural model. The path model was tested through structural equation modeling (SEM) analysis to assess direct and indirect effects. Most results were significant at the *p* < 0.01 level. The empirical results are presented in Table 4.

**TABLE 4:** Path analysis for the model.

Label	Hypotheses	SRW	USRW	*SE*	C.R.	P
H1	BPA → BP	0.195	0.172	0.049	3.536	***
H2	PEV → BP	0.048	0.047	0.066	0.706	0.48
H3	BSR → BP	0.453	0.409	0.082	4.982	***
H4	RNS → BP	0.203	0.18	0.06	2.99	0.003
H5	BPA → BA	0.031	0.029	0.05	0.581	0.561
H6	PEV → BA	0.183	0.191	0.069	2.779	0.005
H7	BSR → BA	0.087	0.084	0.086	0.981	0.327
H8	RNS → BA	0.237	0.225	0.063	3.568	***
H9	BP → BL	0.161	0.176	0.088	1.998	0.046
H10	BP → BA	0.431	0.46	0.082	5.641	***
H11	BA → BL	0.654	0.672	0.096	7.012	***

BPA, brand personality appeal; PEV, perceived emotional value; BSR, brand-based self-realization; RNS, relatedness needs satisfaction; BP, brand passion; BA, brand attachment; BL, brand loyalty; SRW, standardized regression weights; URW, unstandardized regression weights; C.R., critical value; ***p < 0.001.

As indicated in the tables, brand personality appeal has a significant effect on brand passion (β = 0.195, *p* < 0.01), but it does not have a significant effect on brand attachment (β = 0.031, *p* > 0.05). Perceived emotional value does not have a significant effect on brand passion (β = 0.048, *p* > 0.05), but it does have a significant effect on brand attachment (β = 0.183, *p* < 0.01). Brand-based self-realization has a significant effect on brand passion (β = 0.453, *p* < 0.01), but it does not have a significant effect on brand attachment (β = 0.087, *p* > 0.05). Relatedness needs satisfaction has a significant effect on brand passion (β = 0.203, *p* < 0.01) and brand attachment (β = 0.237, *p* < 0.01). Brand passion has a significant effect on brand attachment (β = 0.431, *p* < 0.01) and brand loyalty (β = 0.161, *p* < 0.01), while brand attachment has a significant effect on brand loyalty (β = 0.654, *p* < 0.05).

### Mediating Effects

After establishing the direct relationships, we assessed the mediating effects. The mediate effects of brand passion and brand attachment between brand personality appeal, perceived emotional value, brand-based self-realization, relatedness needs satisfaction, and brand loyalty were tested. This study used the bootstrap approach to calculate mediating effects.

First, brand passion was included as the mediator in the relationship between brand personality appeal, perceived emotional value, brand-based self-realization, relatedness needs satisfaction and brand loyalty. As shown in Table 5, brand passion did not mediate the relationship between brand personality appeal, perceived emotional value, brand-based self-realization, relatedness needs satisfaction and brand loyalty. We then made brand attachment the mediator in the relationship between brand personality appeal, perceived emotional value, brand-based self-realization, relatedness needs satisfaction and brand loyalty. Brand attachment mediated the relationship between perceived emotional value (β = 0.135, *p* < 0.05), relatedness needs satisfaction (β = 0.204, *p* < 0.01) and brand loyalty. Next, brand passion and brand attachment were included as the mediators in the relationship between brand personality appeal, perceived emotional value, brand-based self-realization, relatedness needs satisfaction and brand loyalty. Brand passion and brand attachment mediated the relationship between brand personality appeal (β = 0.051, *p* < 0.01), brand-based self-realization (β = 0.094, *p* < 0.01), relatedness needs satisfaction (β = 0.057, *p* < 0.05) and brand loyalty. Finally, brand attachment was included as the mediator in the relationship between brand passion and brand loyalty. Brand attachment mediated the relationship between brand passion (β = 0.240, *p* < 0.0001) and brand loyalty.

**TABLE 5:** Standardized bootstrap mediated effects test.

	Effect size	*SE*	Bias-corrected 95%			Evidence of
			Lower	Upper	*P*	
BPA → BP → BL	0.036	0.036	0.014	0.132	0.144	Brand passion as mediator
PEV → BP → BL	0.006	0.026	0.022	0.097	0.524	
BSR → BP → BL	0.066	0.06	0.028	0.215	0.142	
RNS → BP → BL	0.04	0.047	0.014	0.168	0.109	
BPA → BA → BL	0.014	0.058	0.087	0.141	0.794	Brand attachment as mediator
PEV → BA → BL	0.135	0.078	0.008	0.313	0.036	
BSR → BA → BL	0.044	0.07	0.103	0.18	0.526	
RNS → BA → BL	0.204	0.068	0.074	0.339	0.006	
BPA → BP → BA → BL	0.051	0.029	0.012	0.137	0.01	Brand passion and brand attachment as mediator
PEV → BP → BA → BL	0.009	0.028	0.038	0.077	0.68	
BSR → BP → BA → BL	0.094	0.04	0.037	0.213	0.002	
RNS → BP → BA → BL	0.057	0.051	0.003	0.201	0.035	
BP → BA → BL	0.24	0.096	0.112	0.479	0.000	Brand attachment as mediator

*BPA, brand personality appeal; PEV, perceived emotional value; BSR, brand-based self-realization; RNS, relatedness needs satisfaction; BP, brand passion; BA, brand attachment; BL, brand loyalty.*

## Discussion

This study examined the impact of idol worship on brand loyalty and explored the mediating effect of brand passion and brand attachment. With empirical statistics, this study found that brand personality appeal, brand-based self-realization, and relatedness needs satisfaction significantly influence brand passion. According to identity theory, brand-idol collaboration gives brands an in-group identity. This dynamic encourages consumers to adopt the brand as part of their self-composition (Das et al., 2019), thus satisfying their inner self-expectations (Zhang and Hung, 2020) and enhancing the brand’s appeal. This type of collaboration also brings fans and idols closer together (Vorderer et al., 2010), which satisfies consumers’ relationship needs (Thomson, 2006) and motivates them to internalize the brand, thus inspiring brand passion (Das et al., 2019).

Another key finding of this study is that perceived emotional value and relatedness needs satisfaction have a significant influence on brand attachment. Idols have special meaning attached to them, and brands that work with these individuals bring out positive emotional experiences, which strengthens positive perceptions of the brand among consumers (Ilicic et al., 2016). Gradually, these positive emotions will turn into more lasting emotional attachments (Vredeveld, 2018), which enhances consumers’ brand attachment.

This study also explored the mediating role of brand passion and brand attachment. Brand personality appeal, brand-based self-realization, and relatedness need satisfaction can influence brand attachment through brand passion and ultimately influence brand loyalty, while perceived emotional value and relatedness needs satisfaction can affect brand loyalty through brand attachment. Moreover, brand passion has a significant influence on brand attachment and brand loyalty, and brand attachment can enhance brand loyalty. These findings align with the results of previous studies (Swimberghe et al., 2014; Bıçakcıoğlu et al., 2018; Kim et al., 2020). As discussed above, brand passion triggers positive and passion-driven behaviors, ultimately leading to brand loyalty (Batra et al., 2012). When fan consumers develop strong emotions for a brand, these feelings encourage them to strengthen their connection with the brand. Gradually, this connection becomes a strong emotional bond, which makes consumers more loyal to certain brands.

The results in this study indicate that brands endorsed by idols exhibit personality appeal and contribute to fan consumers’ brand loyalty, which is consistent with previous research that there is a transfer of personality in the process of brand endorsement (Ang et al., 2006). Another important finding of our study is that the brand carrying the meaning of the idol serves to satisfy the relatedness needs of the fan consumer. Brands with iconic meaning contribute to consumers’ self-fulfillment, which acts as a vital determinant for brand loyalty. And the brand endorsed by the idols contributes to maintaining or enhancing the emotional bond between the fan and their idol. The determinants, such as brand-based self-realization, perceived emotional value, and relatedness needs satisfaction, were rarely mentioned in the literature on celebrity endorsement since these determinants are closely related to the characteristics of idolatry.

## Management Contributions

Enterprises can use idol brand endorsement to enhance the attractiveness of their brand’s personality. When implementing brand marketing strategies, companies can link the personality image of an idol with the brand’s personality image, which will allow the idol’s personality to merge with the brand and give it a more vivid personality performance. This type of collaboration enhances the degree of brand personalization display, improves fan consumers’ positive perception of the brand’s personality, and ultimately heightens the attractiveness of the brand’s personality to fan consumers.

Consumers’ perceived emotional value comes from their brand experience, which centers on the intuitive feelings about the brand that consumers experience through direct contact and brand communication. Therefore, companies can integrate idol elements into their brand as a means of enhancing the emotional experience of fan consumers. By integrating idol elements into all stages of brand presentation and promotion, including product and event design, companies can not only mobilize the interest of fan consumers and stimulate their brand recognition and perceived value, but also enhance their emotional experience of the brand and further strengthen their degree of attachment to the brand.

Idols are vital players that can be used to help fan consumers build relationships with brands. Enterprises can satisfy consumer relatedness needs and narrow the psychological distance between idols and their fans through idol brand endorsement. For these reasons, companies can make efforts to enhance the perceived closeness between their brand and idols. Specifically, brands can conduct brand communication through brand endorsers. Brands can also create close connections with idols through brand promotion activities that involve these celebrity figures. Because fans buy specific products from certain brands to display their love for their idols, brands that are endorsed by these idols enable fans to express these feelings. Fans also imitate the behavior of their idols to achieve their ideal selves, and owning products from brands that are associated with idols is part of their imitation behavior. Therefore, brands that work with idols also provide an outlet for self-realization in the case of fan consumers.

## Limitations and Future Research

One of the limitations of this study is linked to the sample distribution. The majority of the fan consumers that participated in our questionnaire research are young. In reality, this age group is more prone to idolatry and pays more attention to entertainment stars, which meets the research requirements of this study. However, middle-age and elderly individuals also purchase from brands that are linked to their idols, which deserves further research. We also found that most fan consumers in the questionnaire hold a college or graduate-level degree. Therefore, this study does not adequately consider the factors that influence other types of fan consumers’ brand purchasing behaviors. In the future, researchers can expand the scope and diversity of the participant sample to make their studies more comprehensive and objective.

With the growth of social networks, fan interactions are becoming easier and more frequent, and some fans have created their online communities. In these digital spaces, fans interact and even make brand recommendations to one another, which influences their brand preferences. However, it may be that only some fan consumers are influenced by these interactive behaviors, which makes the effects of these types of encounters and connections difficult to measure. To address this issue, future studies can investigate the brand loyalty of fan consumers through in-depth interviews.

## Data Availability Statement

The raw data supporting the conclusions of this article will be made available by the authors, without undue reservation.

## Ethics Statement

Ethical review and approval were not required for the study on human participants in accordance with the local legislation and institutional requirements. The patients/participants provided their written informed consent to participate in this study.

## Author Contributions

LC, SM, and GC contributed in definition of research objectives, developing models, hypotheses, data a nalysis plan, and article writing. LC and SM contributed in data collection, analysis, drawing limitations, future directions, and conclusion of the study. SW contributed to the revision of the manuscript. All authors contributed to the article and approved the submitted version.

## Conflict of Interest

The authors declare that the research was conducted in the absence of any commercial or financial relationships that could be construed as a potential conflict of interest.

## Publisher’s Note

All claims expressed in this article are solely those of the authors and do not necessarily represent those of their affiliated organizations, or those of the publisher, the editors and the reviewers. Any product that may be evaluated in this article, or claim that may be made by its manufacturer, is not guaranteed or endorsed by the publisher.

## Appendix

**TABLE A:** Research variable constructs.

Variable	Title item	Source
Brand personality appeal	The brand has a very attractive personality compared to other brands.	Tho et al., 2016
	The brand’s personality is distinctive compared to other brands.	
	The personality of the brand is easily recognizable compared to other brands.	
Perceived emotional value	I prefer this brand to others.	Hou et al., 2020
	You would prefer this brand to others.	
	This brand makes me feel better than other brands.	
	Compared to other brands, this brand makes me feel better.	
	The quality of the brand is more trustworthy than other brands.	
Brand-based self-realization	The brand brings me closer to my ideal self.	Waterman, 1993; Mandil, 2016
	The brand gives me a stronger sense of involvement.	
	Having the brand gives me a greater sense of fulfillment and satisfaction.	
	The brand gives me a special sense of fit.	
Relationship need satisfaction	I think the brand cares about my feelings.	Chen et al., 2015; Gilal et al., 2020
	I think I have a connection with the brand.	
	I think I am very close to the brand.	
	The brand makes me feel warm.	
Brand passion	I was very excited to see the brand.	Kim et al., 2020; Pourazad et al., 2020
	I aspire to own the brand.	
	Discovering new products from this brand is a pure pleasure for me.	
	I’m happy to use this brand.	
	My relationship with the brand has been very valuable to me.	
Brand attachment	I feel attached to the brand.	Japutra et al., 2018; Garanti, 2019
	I’m passionate about the brand.	
	I think I love the brand.	
	I’m fascinated by the brand.	
	The brand is very much a part of me.	
	The brand automatically evokes fond memories of my past, present and future.	
Brand loyalty	I say positive things about the brand to others.	Hwang et al., 2019
	I would recommend to someone who seeks my advice.	
	I encourage my friends to visit.	
	I intend to visit more often in the future.	
